# An Association Analysis of Anxiety and Psoriasis: Based on the NHANES Database

**DOI:** 10.1002/brb3.70817

**Published:** 2025-09-10

**Authors:** Jie Bai, Yan Ma

**Affiliations:** ^1^ Department of Dermatology Yulin First Hospital Yulin Shaanxi Province China

**Keywords:** anxiety, anxiety days, National Health and Nutrition Examination Survey, psoriasis, restricted cubic spline

## Abstract

**Background:**

Psoriasis is linked with an elevated risk of anxiety disorders, and there may be a temporal relationship between the two. However, the association between anxiety status and its duration with psoriasis is unclear.

**Objectives:**

The present work aimed to figure out the association between anxiety and the risk of psoriasis.

**Methods:**

Data from the National Health and Nutrition Examination Survey (NHANES) 2009–2012 were used. Anxiety state and days of anxiety were applied as the independent variables, and psoriasis as the dependent variable. Weighted logistic regression was employed to analyze the connection between the state and days of anxiety with psoriasis. Restricted cubic spline (RCS) was further utilized to dig out the nonlinear association between days of anxiety and psoriasis. By using weighted logistic regression to further explore the correlation between the combination of anxiety and common cardiovascular risk factors (smoking, hypertension, CVD events) and the risk of psoriasis. Finally, a weighted logistic regression model was constructed for different genders and alcohol consumption subgroups to explore the association between anxiety status and anxiety days and psoriasis and to evaluate the differences in association among different groups.

**Results:**

A total of 8888 participants were included in this project, among whom 265 cases (3.1%) were psoriasis patients. Through the weighted logistics regression model, we observed a significant positive correlation between anxiety (OR: 1.439, 95% CI: 1.008–2.053, *p *= 0.030), number of days with anxiety (OR: 1.018, 95% CI: 1.002–1.033, *p *= 0.014), and the risk of psoriasis in patients. The RCS curve results indicated a linear positive correlation between anxiety days and the risk of psoriasis (*p*‐nonlinear = 0.162). The results of the joint analysis demonstrated that anxiety^−^/smoking^+^ (OR: 1.800, 95% CI: 1.160–2.800, *p *= 0.011), anxiety^+^/smoking^+^ (OR: 2.720, 95% CI: 1.430–5.190, *p *= 0.004), anxiety^+^/hypertension^+^ (OR: 2.010, 95% CI: 1.200–3.370, *p *= 0.011), anxiety^−^/CVD event^+^ (OR: 1.740, 95% CI: 1.080–2.820, *p *= 0.026), and anxiety^+^/CVD event^−^ (OR:1.470, 95% CI: 1.000–2.150, *p *= 0.047) were linked with a significantly elevated risk of psoriasis. The subgroup analysis results showed that women (especially those who drink alcohol) were more sensitive to anxiety status and duration, and the association between increased anxiety days and increased risk of psoriasis was more significant, while no similar significant association was observed in men.

**Conclusion:**

Anxiety and the number of days with anxiety are positively linked with the risk of psoriasis. Individuals with anxiety^+^/smoking^+^ or anxiety^+^/hypertension^+^ have a higher risk of developing psoriasis. We recommended that, in the prevention and management of psoriasis, individuals need to cope with stress to alleviate anxiety symptoms and try not to smoke. Women should pay special attention to regulating anxiety while drinking alcohol and monitoring blood pressure and cardiovascular health regularly.

## Graphical abstract

Anxiety and higher anxiety days significantly increase psoriasis risk, with synergistic effects observed when combined with smoking or hypertension. Notably, females with alcohol consumption exhibit heightened sensitivity to anxiety‐related psoriasis risk.

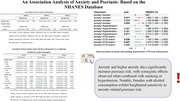

## 1 Introduction

Psoriasis is a chronic inflammatory skin disease characterized by silver scales on the skin covering well‐defined red patches, commonly found on the scalp, limbs, and sacral region (Nair and Badri 2024). The disease is mainly a T lymphocyte‐mediated autoimmune disease, in which activated T cells drive the activation of the IL‐23/17 cell axis in the skin, resulting in inflammation in keratinocytes and induction of epidermal hyperplasia and recruitment of the leukocyte subset, thereby causing the occurrence and development of psoriatic plaques (Hawkes et al. 2017). Psoriasis affects approximately 55.8 million adults worldwide, with the largest number of adults affected (approximately 3.4 million) in the United States, imposing a heavy burden on global public health (Parisi et al. 2020). Therefore, figuring out possible methods for preventing and managing psoriasis is urgent.

The appearance of psoriasis often makes patients feel inferior, seriously affecting their quality of life and leading to psychological disorders such as anxiety. In turn, anxiety exacerbates psoriasis, thus creating a vicious cycle (Kouris et al. 2017). A tight link between psoriasis and an elevated risk of anxiety (*β*: 3.8, 95% confidence interval [CI]: 1.8–5.7, *p *< 0.001) has been discovered (Soliman 2021). There may be a temporal dynamic association between psoriasis and increased risk of anxiety (Hunter et al. 2013), and cross‐sectional evidence suggests that the severity and duration of psoriasis are positively correlated with anxiety (Lakshmy et al. 2015), providing a basis for the hypothesis regarding anxiety as a trigger for psoriasis. The previous Mendelian randomization (MR) study revealed no association between psoriasis and the risk of anxiety. However, MR studies were based on European ancestry populations, and the universality of their results may be limited by racial genetic differences (M. Chu et al. 2023; Wang et al. 2023). Therefore, there is currently a lack of quantitative research on the relationship between anxiety states/duration (such as anxiety days) and psoriasis risk in other populations, such as multiethnic Americans. In addition, smoking, hypertension, and cardiovascular disease (CVD) are clear independent risk factors for psoriasis (Kamiya et al. 2019; Lu et al. 2013). Mechanistically, these factors may have a synergistic effect with anxiety. Smoking exacerbates anxiety by activating the hypothalamic–pituitary–adrenal (HPA) axis through nicotine (LaFond et al. 2024; Y. Liu, Cao, et al. 2022). Hypertension and anxiety share the sympathetic activation pathway (Hasebe et al. 2024; Shao et al. 2023), and CVD events can induce psychological stress (Khan et al. 2025). These three factors may participate in the pathogenesis of psoriasis by promoting systemic inflammation (such as cytokine release of IL‐17, TNF‐α, etc.) and immune dysregulation (such as immune cell activation and changes in gut microbiota), forming an interactive network (Andujar et al. 2022; Laguardia et al. 2024; Potestio et al. 2024). However, there is currently no research exploring the combined effect of anxiety and these risk factors on psoriasis.

Given this, this study utilized the National Health and Nutrition Examination Survey (NHANES) database in the United States in a sequent analysis, which has the advantage of including multiethnic populations and can supplement the limitations of genetic research in Europe. In addition, collecting anxiety frequency data through a standardized question system provides a unique perspective for exploring the dose‐response of anxiety duration. This study used self‐reported anxiety days as an exposure indicator. Although it is not a structured clinical diagnostic tool, this indicator has been included in the CDC's core monitoring system for health‐related quality of life (HRQoL) (Andresen et al. 2003), which can effectively reflect the frequency of psychological distress in the population. Therefore, this study hypothesized that self‐reported anxiety frequency (status/days) is associated with psoriasis risk. This study further explored its combined effects with smoking, hypertension, and CVD events, as well as its association in different gender and alcohol consumption subgroups, aiming to provide a theoretical basis for developing psoriasis prevention strategies targeting psychological stress and multiple risk factors.

## 2 Methods

### 2.1 Data Source and Study Population

NHANES is a cross‐sectional survey study conducted by the National Center for Health Statistics (NCHS) to collect information on the health and nutritional status of adults and children in the United States, with data collected every 2 years. The NHANES data have been approved by the NCHS Research Ethics Review Board. All participants signed informed consent forms before participating in the survey. NHANES survey data are publicly available online at https://www.cdc.gov/nchs/nhanes/index.htm.

**FIGURE 1:**
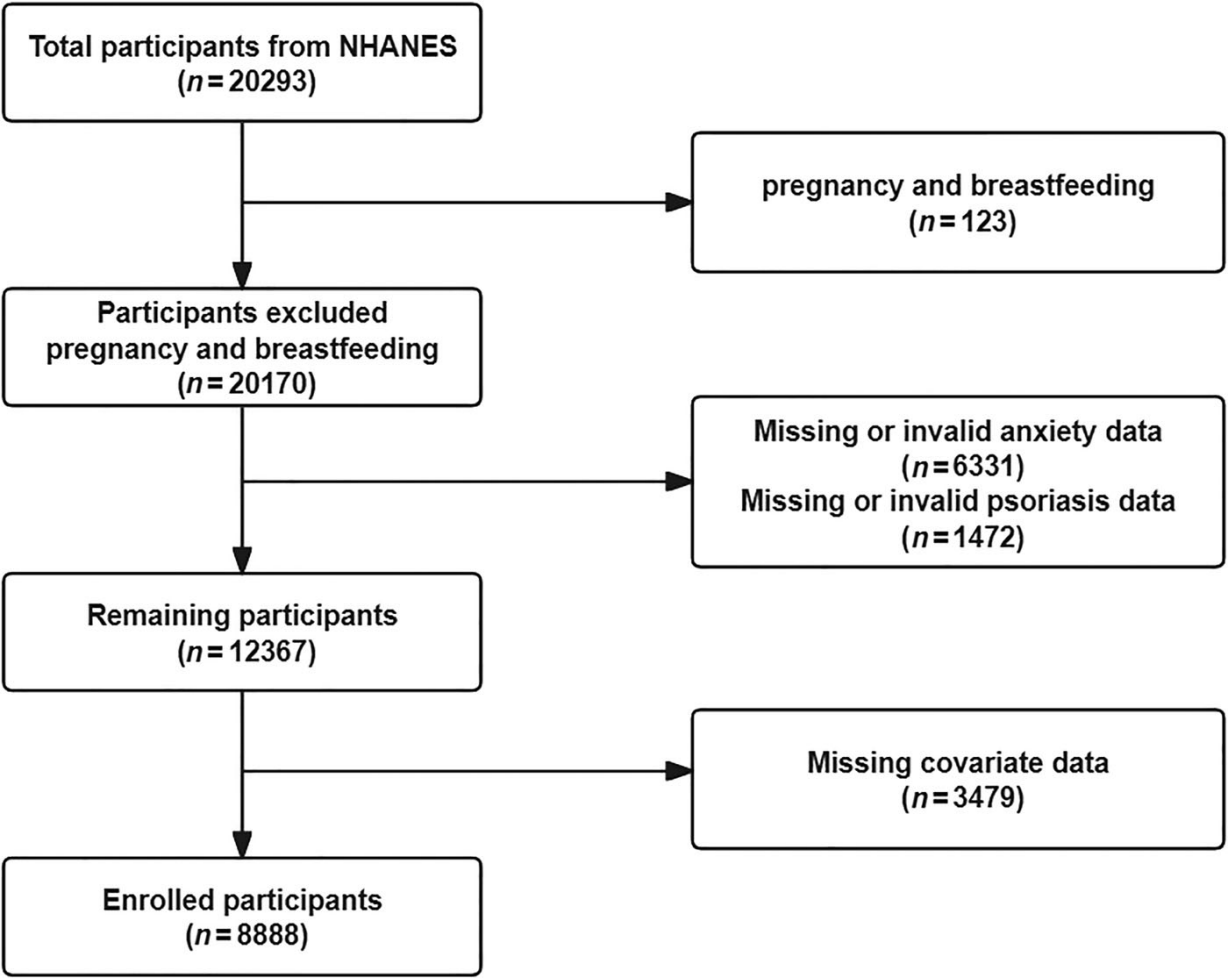
The screening flowchart of the studied population.

We utilized data from the NHANES for a total of two cycles from 2009–2010 (published in September 2011) (National Center for Health Statistics 2011) and 2011–2012 (updated in January 2015) (National Center for Health Statistics 2015), which included a total of 20,293 participants. We excluded participants who were pregnant and breastfeeding (*n* = 123), participants with missing or unavailable data on anxiety symptoms (*n* = 6331) and psoriasis data (*n* = 1472), as well as other participants with missing data (*n* = 3479), resulting in a total of 8888 participants. The specific screening process for the population is described in Figure 1.

### 2.2 Exposure Variable: Anxiety

During data collection, interviewers used a computer‐assisted personal interview system at the Mobile Screening Center to collect information from participants about their anxiety states through questionnaires. During the personal interview, anxiety was examined by asking questions: approximately how many days in the past 30 days have you felt worried, nervous, or anxious? Anxiety status was categorized as “yes” (feeling anxious 7–30 days per month) and “no” (feeling anxious 0–6 days per month) based on the number of anxious days (Dantzer and Keet 2020). The classification criteria for anxiety state refer to the HRQoL‐14 monitoring guidelines (Dantzer and Keet 2020) of the Centers for Disease Control and Prevention (CDC) in the United States and have been widely used in NHANES research (Dantzer and Keet 2020; Li et al. 2025), with a reliable methodological basis. The CDC Health Day Measurement Scale is designed specifically for rapid screening of populations. This binary method balances sensitivity and specificity, making it easier to reveal the public health burden of anxiety exposure and compare it with previous studies. The HRQoL assessment has moderate to excellent monitory reliability (Andresen et al. 2003; Wen et al. 2024).

### 2.3 Outcome Variable: Psoriasis

The definition of psoriasis in this study was based on a standardized questionnaire evaluation in the NHANES database, specifically determined by the following question: “Have you ever been informed by a doctor or other healthcare professional that you have psoriasis?” The “yes” answer indicated psoriasis, while the “no” answer indicated “non‐psoriasis.” This definition method relies on the diagnostic records of medical professionals and does not require further grading. It has been widely used in large‐scale population epidemiological surveys, and its effectiveness has been validated in previous psoriasis‐related studies based on NHANES (Ruan et al. 2022).

### 2.4 Variables

The covariates included in this project were age, race, alcohol consumption, body mass index (BMI), gender, smoking, diabetes, hypertension, physical activity (PA), and CVD history.

Races included Mexican American, non‐Hispanic Black and White, other Hispanic, and other. The BMI classification was as follows: underweight/healthy weight: ≤ 25 kg/m^2^, overweight: 25–30 kg/m^2^, or obese: > 30 kg/m^2^ (Guo et al. 2022). Alcohol consumption was defined as follows: ≥ 12 drinks/year, a drink refers to 12 oz. of beer, a 5 oz. glass of wine, or one and a half ounces of liquor). Smoking status was categorized as never (< 100 cigarettes in lifetime), former (≥ 100 cigarettes in lifetime, now no smoking at all), and current (≥ 100 cigarettes in lifetime, still smoking) (Gui et al. 2023). PA was assessed by summing the number of minutes of activity per week multiplied by the Metabolic Equivalent of Task (MET) score for each activity (N. M. Chu et al. 2022). The MET was a measure of PA intensity and was based on the NHANES guidelines that assigned a metabolic equivalent score to each activity on the following scale: 8 points for vigorous work‐related activities and 4 points for moderate work‐related activities. PA < 600 min/week indicated low‐level PA. PA between 600 and 8000 min/week indicated a moderate level. PA ≥ 8000 min/week was considered a high level (Yuan et al. 2024). Diagnostic Criteria for Diabetes: (1) affirmative answer to the question “Have you been told by a doctor or health professional that you have diabetes?” and (2) affirmative answer to the question “Have you used antidiabetic medications?” (3) Glycosylated hemoglobin (HbA1c) ≥ 6.5% (4) Fasting blood glucose (FPG) ≥ 126 mg/dL (Zhao and Li 2022). The diagnostic criteria for hypertension were defined by affirmative answers to the following questions: “Have you been told by a doctor or other health professional that you have hypertension?”; “Are you currently taking prescription medication for hypertension?”; Or systolic blood pressure ≥ 140 mmHg or diastolic blood pressure ≥ 90 mmHg (Kan et al. 2024). The history of CVD was determined based on an affirmative answer to any of the following questions: “Have you been told by a doctor or other health professional that you have had a heart attack/stroke/congestive heart failure/angina/coronary heart disease?” (Zhang et al. 2018).

### 2.5 Statistical Analysis Methods

A standard baseline table was drawn using the *tableone* package (Tableone: Create “Table 1” to Describe Baseline Characteristics With or Without Propensity Score Weights n.d.), which grouped the study population according to the disease status of psoriasis and systematically displayed the distribution differences of demographic characteristics, lifestyle factors, and clinical indicators. Respondents were grouped according to the total population characteristics as to whether they had psoriasis or not. Categorical variables in the statistical analysis were expressed as frequency and percentage (*n* [%]), and continuous variables were expressed as mean and standard deviation, providing a basic reference for population characteristics for subsequent analysis. Multivariate weighted logistic regressions were constructed using the *survey* package (Survey Snalysis in R n.d.) to probe into the linkage between the status and days of anxiety with psoriasis in the three models. This package is suitable for complex sampling in NHANES databases, including stratified and cluster sampling. By weighted adjustment, this package can correct sampling bias, make sample characteristics closer to the general population in the United States, ensure the representativeness and reliability of multivariate weighted logistic regression analysis results, and accurately estimate the odds ratio (OR) and 95% confidence interval (CI) associated with anxiety and psoriasis.

This study constructed three models, with Model 1 without adjustment as the basic reference, directly presenting the uncorrected original association between anxiety (anxiety days, anxiety state) and psoriasis, facilitating the observation of initial association trends. Model 2 adjusted for age, gender, race, BMI, alcohol consumption, smoking, and PA. These factors are recognized as basic confounding factors, and prioritizing the correction of these core factors can reduce the interference of basic confounding on association estimation. Model 3 further adjusted the history of diabetes, hypertension, and CVDs on the basis of Model 2. These variables are important chronic diseases and cardiovascular‐related factors and are closely related to inflammatory reactions and immune disorders. They may interact with anxiety and psoriasis through a common pathological mechanism. After supplementary adjustment, residual confounding can be further controlled so that the correlation estimation is closer to the real effect.

A restricted cubic spline (RCS) was applied to test the potential nonlinear association of anxiety days with psoriasis. RCS can flexibly and robustly capture the nonlinear relationship between continuous variables and outcome events. Compared with simple linear regression, which can only reveal the linear correlation between variables or the node setting bias that may exist in segmented linear models, RCS can more accurately reflect the dynamic correlation pattern between anxiety days from 0 to 30 days and psoriasis risk by fitting a smooth curve with a small number of preset nodes (knots), while avoiding overfitting (B. Liu et al. 2023).

We combined anxiety status (binary variables: “no” = 0–6 days of anxiety per month, “yes” = 7–30 days of anxiety per month) with each risk factor to form four types of joint exposure groups: (1) anxiety^−^/risk factor—(no anxiety and no risk factor); (2) anxiety^−^/risk factors^+^(without anxiety but with the risk factor); (3) anxiety^+^/risk factors—(with anxiety but without the risk factor); (4) anxiety^+^/risk factors^+^(with both anxiety and risk factors). The forest plots were plotted to visually display the OR values, 95% CI, and *p* values of each combined exposure group, clearly demonstrating the differences in the impact of different combinations on the risk of psoriasis. Finally, we constructed three weighted logistic regression models for different genders and alcohol consumption subgroups and calculated the OR and 95% CI of anxiety status and anxiety days with psoriasis risk to evaluate the differences in association among different groups. All statistical analyses were conducted using R software (V4.3.3), with *p *< 0.05 considered significant.

## 3 Result

### 3.1 Baseline Characteristics of Participants

This project included 8,888 participants, among whom there were 265 patients with psoriasis, accounting for 3.1%. The baseline characteristics of the population are displayed in Table 1. The average age of all participants was 47.40 ± 16.83 years, with 4419 females (50.4%) and 2358 individuals (26.8%) experiencing symptoms of anxiety. Individuals with psoriasis and non‐psoriasis exhibited significant differences in race, smoking, history of CVD, hypertension, and anxiety (all *p* < 0.05). Compared to non‐psoriasis patients, psoriasis patients had higher proportions of non‐Hispanic white individuals (79.9%), past smokers (38.3%), a history of CVD (14.7%), hypertension (61.2%), and anxiety (34.4%).

**TABLE 1:** The Baseline Characteristics of NHANES Participants in 2009–2012.

Variable	Total	Non‐psoriasis	Psoriasis	*p* value
Overall	8888	8623 (96.9)	265 (3.1)	
Age	47.40 (16.83)	47.32 (16.86)	49.68 (15.65)	0.070
Gender				0.227
Male	4469 (49.6)	4328 (49.5)	141 (53.6)	
Female	4419 (50.4)	4295 (50.5)	124 (46.4)	
Race				0.002
Mexican American	1181 (7.3)	1155 (7.4)	26 (4.9)	
Other Hispanic	859 (5.4)	831 (5.4)	28 (5.1)	
Non‐Hispanic White	4068 (70.2)	3912 (69.9)	156 (79.9)	
Non‐Hispanic Black	1897 (10.6)	1865 (10.7)	32 (6.1)	
Other race	883 (6.6)	860 (6.6)	23 (4.0)	
BMI (kg/m^2^)				0.223
< 25	2627 (30.5)	2563 (30.7)	64 (24.0)	
25–30	2931 (33.7)	2846 (33.6)	85 (34.2)	
> 30	3330 (35.8)	3214 (35.7)	116 (41.8)	
Alcohol				0.784
No	2319 (20.4)	2254 (20.4)	65 (19.7)	
Yes	6569 (79.6)	6369 (79.6)	200 (80.3)	
Smoking				<0.001
Never	4858 (55.2)	4746 (55.7)	112 (41.0)	
Past	2161 (24.9)	2077 (24.5)	84 (38.3)	
Now	1869 (19.9)	1800 (19.9)	69 (20.8)	
Activity scores (min/week)				0.948
< 600	3503 (34.7)	3401 (34.7)	102 (33.8)	
600–8000	4283 (52.5)	4153 (52.5)	130 (52.7)	
≥ 8000	1102 (12.8)	1069 (12.8)	33 (13.5)	
Diabetes				0.255
No	7432 (88.0)	7220 (88.1)	212 (85.3)	
Yes	1456 (12.0)	1403 (11.9)	53 (14.7)	
History of CVD				0.001
No	7973 (91.9)	7757 (92.1)	216 (85.3)	
Yes	915 (8.1)	866 (7.9)	49 (14.7)	
Hypertension				0.008
No	4116 (50.4)	4012 (50.7)	104 (38.8)	
Yes	4772 (49.6)	4611 (49.3)	161 (61.2)	
Anxiety				0.023
No	6530 (73.2)	6359 (73.4)	171 (65.6)	
Yes	2358 (26.8)	2264 (26.6)	94 (34.4)	

*Note*: n (%) represented the categorical variable, and mean (SD) represented the continuous variable. *n* was unweighted. *n* (%), mean, and SD were weighted.

Abbreviations: BMI, body mass index; CVD, cardiovascular disease.

### 3.2 Association Between Anxiety and Psoriasis

Table 2 presents the results of the weighted logistic regression model on the association between days and the status of anxiety and psoriasis. In Model 3, adjusting for all confounding factors, the number of anxious days was positively linked with psoriasis (OR = 1.018, 95% CI: 1.002–1.033, *p *= 0.014). Additionally, in all three models, the risk of psoriasis in individuals with anxiety symptoms was significantly higher compared to those without anxiety symptoms (OR > 1, *p *< 0.05).

**TABLE 2:** Associations between anxiety and psoriatic.

	Model 1		Model 2		Model 3	
	OR [95% CI]	*p*	OR [95% CI]	*p*	OR [95% CI]	*p*
Anxiety days	1.018 [1.005, 1.032]	0.006	1.019 [1.004, 1.034]	0.008	1.018 [1.002, 1.033]	0.014
Anxiety status						
Non‐anxiety	1 [Reference]		1 [Reference]		1 [Reference]	
Anxiety	1.452 [1.053, 2.003]	0.018	1.470 [1.038, 2.083]	0.021	1.439 [1.008, 2.053]	0.030

*Note*: Model 1 with no adjustment; Model 2 adjusted for age, sex, race, BMI, alcohol consumption, smoking, and PA; and Model 3 adjusted for age, sex, race, BMI, alcohol consumption, smoking, PA, diabetes, hypertension, and history of CVD.

Abbreviations: BMI, body mass index; CI, confidence interval; CVD, cardiovascular disease; OR, odds ratio.

### 3.3 Nonlinear Association Between Days of Anxiety and Psoriasis

**FIGURE 2:**
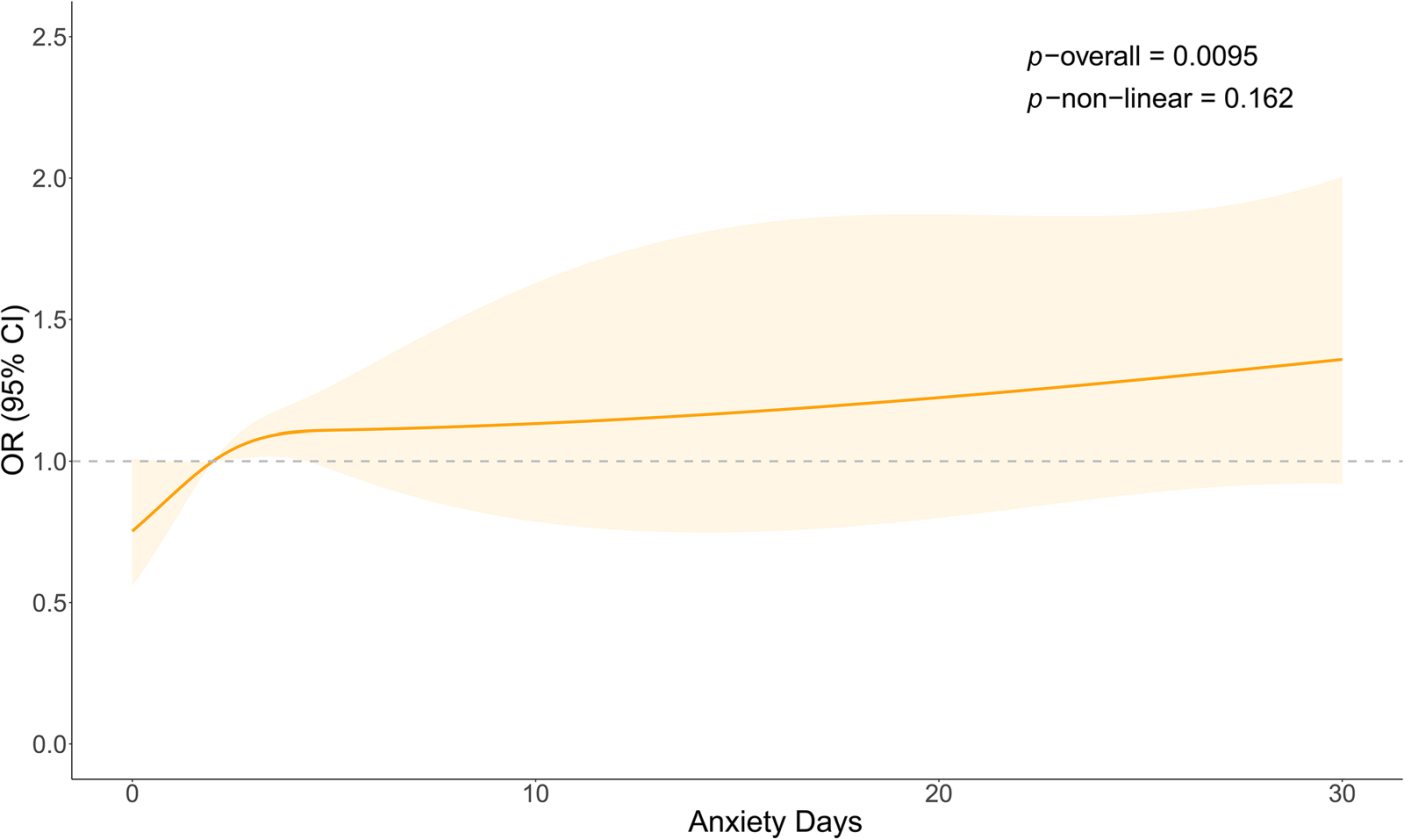
The OR of anxiety days and psoriasis adjusted by covariates (NHANES 2009–2012). The RCS line is adjusted for age, race, gender, BMI, smoking, alcohol, hypertension, diabetes, and history of CVD. The OR is represented by the orange line, and the shaded part represents the 95% CI. BMI, body mass index; CI, confidence interval; CVD, cardiovascular disease; OR, odds ratio.

In the weighted logistic regression model adjusting for all confounding factors, the relationship between the days of anxiety and the risk of psoriasis was dissected by using RCS (Figure 2). The results indicated a significant overall trend between the days of anxiety and the risk of psoriasis (*p* overall = 0.0095), with a great increase in the risk of psoriasis as the number of anxiety days per month increased. There was a linear relationship between days of anxiety and the risk of psoriasis (*p*‐nonlinear = 0.162).

### 3.4 Combined Effects of Anxiety and Common Cardiovascular Risk Factors (Smoking, Hypertension, and CVD) on Psoriasis

**FIGURE 3:**
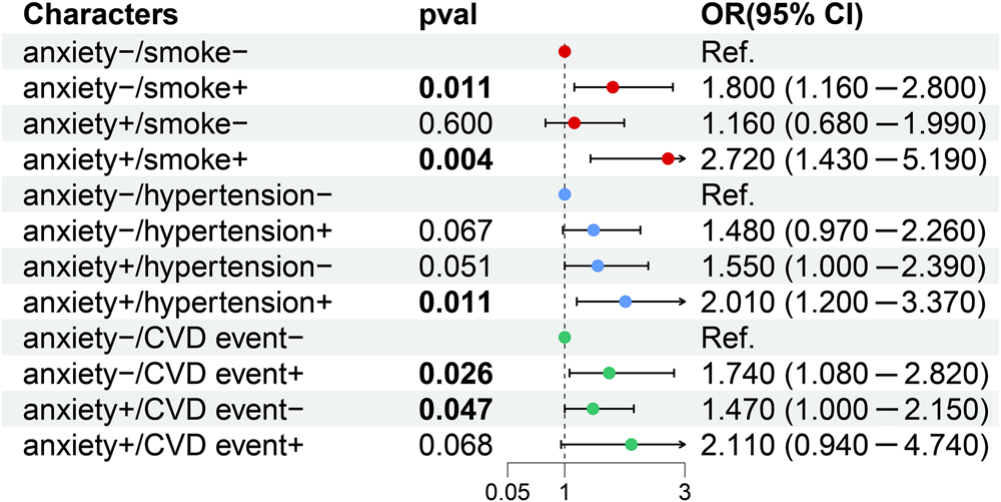
Joint association of anxiety and smoking, hypertension, and CVD events with psoriasis.

We analyzed the risk of psoriasis prevalence in association with common cardiovascular risk factors (smoking, hypertension, and CVD) for the respondents’ anxiety status. As shown in Figure 3, compared to the normal population (anxiety^−^/smoking^−^), individuals with anxiety^−^/smoking^+^ and anxiety^+^/smoking^+^ possessed a positive linkage with the risk of psoriasis, with corresponding OR values and 95% CIs of 1.800 (1.160–2.800, *p *= 0.011) and 2.720 (1.430–5.190, *p *= 0.004), respectively. Anxiety^+^/hypertension^+^ individuals had a significantly elevated risk of psoriasis (OR: 2.010, 95% CI: 1.200–3.370, *p *= 0.011). Additionally, individuals with anxiety^−^/CVD event^+^ and anxiety^+^/CVD event^−^ had a considerably elevated risk of psoriasis, with corresponding OR values and 95% CIs of 1.740 (1.080–2.820, *p *= 0.026) and 1.470 (1.000–2.150, *p *= 0.047), respectively. Individuals who were also anxiety^+^/CVD event^+^ had a significantly elevated OR (OR = 2.110) despite this association not being significant (*p *= 0.068).

### 3.5 Association Analysis Between Anxiety and Psoriasis in Gender Stratification and Alcohol Consumption Subgroups

To explore the potential impact of gender on the association between anxiety and psoriasis, this study conducted gender‐stratified analysis and further subgroup analysis in combination with alcohol consumption status. The results of Table 3 showed that the association between anxiety status and psoriasis did not reach statistical significance in both males and females among different adjustment models (*p *> 0.05). For anxiety days, women showed a significant positive correlation in the unadjusted model (OR = 1.025, 95% CI: 1.003–1.047, *p * = 0.021). Although the correlation weakened in Model 3, it still showed a significant trend (OR = 1.022, 95% CI: 0.995–1.049, *p * = 0.086). However, no significant association was observed in males among all models (*p *> 0.05).

**TABLE 3:** Associations between anxiety (status and days) and psoriasis stratified by sex.

	Model1		Model2		Model3	
	OR [95% CI]	*p*	OR [95% CI]	*p*	OR [95% CI]	*p*
Anxiety status						
Male	1.336 (0.782–2.283)	0.271	1.373 (0.792–2.381)	0.229	1.364 (0.781–2.383)	0.242
Female	1.638 (0.944–2.843)	0.068	1.569 (0.881–2.797)	0.104	1.511 (0.835–2.735)	0.144
Anxiety days						
Male	1.013 (0.991–1.036)	0.241	1.015 (0.992–1.038)	0.188	1.014 (0.991–1.038)	0.200
Female	1.025 (1.003–1.047)	0.021	1.024 (0.998–1.050)	0.054	1.022 (0.995–1.049)	0.086

*Note*: Model 1 with no adjustment; Model 2 adjusted for age, sex, race, BMI, alcohol consumption, smoking, and PA; and Model 3 adjusted for age, sex, race, BMI, alcohol consumption, smoking, PA, diabetes, hypertension, and history of CVD.

Abbreviations: BMI, body mass index; CI, confidence interval; CVD, cardiovascular disease; OR, odds ratio.

After further stratification by alcohol consumption status (Table 4), it was found that anxiety status was significantly positively correlated with psoriasis in the drinking population (Model 3: OR = 1.742, 95% CI: 1.246–2.435, *p *= 0.001), and the association was more significant in the female subgroup (Model 3: OR = 2.100, 95% CI: 1.046–4.215, *p *= 0.025), while the male subgroup did not reach a significant level (Model 3: OR = 1.512, 95% CI: 0.842–2.716, *p *= 0.138). In the drinking population, the association between anxiety days and psoriasis was also significant (Model 3: OR = 1.024, 95% CI: 1.010–1.039, *p *= 0.001) and remained significant in the female subgroup after complete adjustment (Model 3: OR = 1.032, 95% CI: 1.002–1.064, *p *= 0.027), while no such association was observed in the male subgroup (Model 3: OR = 1.018, 95% CI: 0.994–1.042, *p *= 0.122). In addition, among nondrinkers, the association between anxiety status and anxiety duration with psoriasis did not reach a significant level in both males and females (*p *> 0.05).

**TABLE 4:** Associations between anxiety (status and days) and psoriasis in sex subgroups stratified by alcohol consumption.

	Model1		Model2		Model3	
	OR [95% CI]	*p*	OR [95% CI]	*p*	OR [95% CI]	*p*
Anxiety status						
Alcohol	1.730 [1.265, 2.366]	**< 0.001**	1.759 [1.261, 2.455]	**< 0.001**	1.742 [1.246, 2.435]	**0.001**
Male	1.429 [0.813, 2.511]	0.198	1.521 [0.850, 2.719]	0.133	1.512 [0.842, 2.716]	0.138
Female	2.280 [1.184, 4.389]	**0.010**	2.183 [1.113, 4.284]	**0.016**	2.100 [1.046, 4.215]	**0.025**
Non‐alcohol	0.579 [0.268, 1.253]	0.149	0.507 [0.227, 1.131]	0.078	0.477 [0.209, 1.087]	0.059
Male	0.769 [0.130, 4.536]	0.763	0.536 [0.077, 3.736]	0.503	0.502 [0.062, 4.053]	0.488
Female	0.522 [0.215, 1.263]	0.134	0.487 [0.188, 1.261]	0.115	0.450 [0.172, 1.180]	0.082
Anxiety days						
Alcohol	1.024 [1.010, 1.038]	**< 0.001**	1.025 [1.011, 1.040]	**< 0.001**	1.024 [1.010, 1.039]	**0.001**
Male	1.015 [0.991, 1.040]	0.196	1.018 [0.994, 1.042]	0.118	1.018 [0.994, 1.042]	0.122
Female	1.036 [1.011, 1.062]	**0.003**	1.034 [1.005, 1.065]	**0.015**	1.032 [1.002, 1.064]	**0.027**
Non‐alcohol	0.991 [0.955, 1.028]	0.617	0.987 [0.949, 1.027]	0.497	0.983 [0.943, 1.025]	0.393
Male	0.999 [0.918, 1.087]	0.977	0.979 [0.892, 1.074]	0.627	0.976 [0.882, 1.080]	0.610
Female	0.988 [0.956, 1.021]	0.458	0.986 [0.951, 1.023]	0.428	0.982 [0.944, 1.021]	0.321

*Note*: Model 1 with no adjustment; Model 2 adjusted for age, sex, race, BMI, alcohol consumption, smoking, and PA; and Model 3 adjusted for age, sex, race, BMI, alcohol consumption, smoking, PA, diabetes, hypertension, and history of CVD. Values in bold represent significant statistical significance.

Abbreviations: BMI, body mass index; CI, confidence interval; CVD, cardiovascular disease; OR, odds ratio.

## 4 Discussion

This project probed into the linkage between status and days of anxiety and psoriasis based on two cycles of data from the NHANES database from 2009–2012, further assessed the potential nonlinear association between days of anxiety and psoriasis, and finally examined the effect of the association between anxiety and common cardiovascular risk factors (smoking, hypertension, and CVD) combined on psoriasis. The results uncovered that anxiety and the number of anxious days were positively linked with the risk of psoriasis, with a linear relationship between the number of anxious days and the risk of psoriasis. The joint analysis manifested that anxiety^−^/smoking^+^, anxiety^+^/smoking^+^, anxiety^+^/hypertension^+^, anxiety^−^/CVD event^+^, and anxiety^+^/CVD event^−^ were positively linked with the risk of psoriasis. The association analysis between anxiety and psoriasis in gender stratification and alcohol consumption subgroups showed that women (especially those who drink alcohol) were more sensitive to anxiety and anxiety duration, with a more significant association between increased anxiety days and increased psoriasis risk. No similar significant association was observed in men.

Several previous studies are centered on the relationship between psoriasis (exposure variable) and the prevalence of anxiety (outcome variable). According to a systematic evaluation study, the prevalence of anxiety in psoriasis patients ranged from 7% to 48%, which was higher than in healthy controls (OR: 2.91, 95% CI: 2.01–4.21, *p *< 0.001). The treatment of psoriasis can improve anxiety symptoms (Fleming et al. 2017). Another study on a British population noted that patients with psoriasis have a significant 1.23‐fold increased risk of anxiety compared to healthy patients (*p *< 0.05). However, fewer studies dissect the relationship between anxiety (exposure variable) and the prevalence of psoriasis (outcome variable). Only one systematic evaluation has probed into a possible temporal linkage between psychological stress and the onset, recurrence, and severity of psoriasis (Stewart et al. 2018). Therefore, this paper focused on exploring the relationship between the status and duration of anxiety and the risk of psoriasis.

This study provides important epidemiological evidence for anxiety (state and duration) as a risk factor for psoriasis. We speculate that chronic low‐grade inflammation is a key biological bridge connecting anxiety and psoriasis, with HPA axis dysfunction and the IL‐17‐mediated immune pathway playing a central role. An anxiety state can lead to abnormal HPA axis function. Under acute stress, the HPA axis activates and releases cortisol to suppress inflammatory responses. However, chronic anxiety may lead to HPA axis dysfunction (insufficient cortisol secretion or rhythm disorders) and/or decreased sensitivity to glucocorticoid receptors (Cassiers et al. 2025; Kanes et al. 2023; Li et al. 2025; Ring 2025). This HPA axis imbalance weakens the body's inhibitory effect on inflammation. At the same time, the sympathetic nervous system, activated by anxiety, releases neurotransmitters such as norepinephrine, which can directly act on adrenergic receptors on the surface of immune cells (Choi et al. 2024; Cox 2024; Won and Kim 2020). The dysfunction of the HPA axis and overactivation of the sympathetic nervous system jointly lead to immune system imbalance, especially promoting the differentiation and expansion of pro‐inflammatory T helper cell 17 (Th17) and driving the upregulation of the IL‐17 axis (Chen et al. 2023; Marek‐Jozefowicz et al. 2022; Potestio et al. 2024). IL‐17 is the core cytokine in the pathogenesis of psoriasis. It acts on keratinocytes, inducing their excessive proliferation, abnormal differentiation, and releasing a large amount of other pro‐inflammatory factors (such as TNF‐α, IL‐6, and IL‐8) and antimicrobial peptides, forming a self‐amplifying inflammatory loop, ultimately leading to characteristic epidermal hyperplasia and inflammatory infiltration in psoriasis (Akazawa et al. 2023; Gao et al. 2025; Hawkes et al. 2017; Potestio et al. 2024). Therefore, anxiety may increase susceptibility to psoriasis or worsen its condition by disrupting the HPA axis neuroendocrine immune network, amplifying the inflammatory cascade driven by IL‐17. In addition, reduced brain‐derived neurotrophic factor (BDNF) signal transduction is also believed to indirectly act on immune cells and keratinocytes by activating the inflammatory factor NF‐κB during anxiety and participate in the development of psoriasis (Lima Giacobbo et al. 2019; Sochal et al. 2022; Zhou et al. 2022). Although this study is a cross‐sectional design and cannot establish a causal timeline, relevant prospective studies support the view that anxiety/stress may be a trigger or risk factor for psoriasis. A study based on Swedish male conscription queue data found a significant correlation between adolescent low stress adaptation ability and increased risk of subsequent psoriasis (Laskowski et al. 2024). The mentioned study is a prospective study using standardized stress assessment tools, indicating that psychological vulnerability may occur before psoriasis. These pieces of evidence are consistent with the findings of this study, collectively suggesting that managing anxiety/stress may have potential value in preventing psoriasis.

In joint analysis, we first found that the risk of psoriasis in patients was positively linked with anxiety^−^/smoking^+^. Armstrong et al. (2014) put forward that smoking is an independent risk factor for the development of psoriasis, with current smoking and past smoking associated with increased incidence of psoriasis (ORs of 1.78 and 1.62, respectively, *p *< 0.05), which was in line with our results. In addition, individuals who were anxiety^+^/smoking^+^ had a considerably elevated risk of developing psoriasis. Regular smoking correlates with an elevated risk of developing new‐onset anxiety disorders (OR > 1, *p *< 0.001) (Mojtabai and Crum 2013), proving that smoking heightens the risk of psoriasis by triggering anxiety. A possible explanation for the combined effect between the two is that smoke produced by smoking contains high levels of free radicals and toxic substances that can cause oxidative stress and systemic inflammation and secretion of pro‐inflammatory cytokines, which trigger and exacerbate symptoms of anxiety through a variety of pathways (e.g., immune and HPA axes), amplify inflammatory responses, and recruit immune‐inflammatory cells to infiltrate the dermis, causing psoriasis (Hahad et al. 2021; Tong et al. 2022). In addition, patients with anxiety^+^/hypertension^+^ had a significant positive link with the risk of psoriasis. A cross‐sectional study uncovered that hypertension and increased anxiety symptoms were greatly associated (OR: 4.9, 95% CI: 2.75–8.82, *p *< 0.001) (Namdar et al. 2024). We speculated that the combination of anxiety and hypertension may significantly elevate the risk of psoriasis by exacerbating individual anxiety symptoms. This phenomenon may be explained by the fact that individuals with hypertension often have elevated systemic inflammatory responses, which can activate microglia cells to further release pro‐inflammatory mediators. Elevated levels of cytokines can affect changes in the frontal and limbic structures of the brain, exacerbating anxiety symptoms and significantly increasing the risk of psoriasis (Won and Kim 2020; Xiao and Harrison 2020). Finally, individuals with anxiety^−^/CVD event^+^ were linked with an elevated risk of psoriasis. An MR analysis manifested that coronary artery disease increases the risk of psoriasis (OR: 1.11, *p *< 0.05), which supports our research findings. The occurrence and development of CVD and the risk of psoriasis may involve inflammation and immunity (Wang et al. 2022). Atherosclerosis, the main cause of CVD, is closely related to systemic inflammatory response, and the inflammatory mediators it releases may cause skin barrier alteration and infiltration of skin immune cells, which facilitates hyperproliferation of keratinocytes and alters their differentiation status, consequently resulting in psoriatic plaques (C. Liu, Chen, et al. 2022; Sabat et al. 2019; Wolf and Ley 2019). These combined effects highlight the central role of the inflammatory network in integrating psychological stress and traditional risk factors to promote psoriasis. Intervention strategies should simultaneously focus on mental health (alleviating anxiety) and controlling cardiovascular risk factors (quitting smoking, lowering blood pressure, regulating fat, and managing CVD) to minimize the risk of psoriasis. Individuals with anxiety symptoms, smoking, or hypertension should be considered high‐risk groups for psoriasis. It is recommended that dermatologists and general practitioners actively screen and regularly monitor the skin condition of high‐risk individuals during diagnosis and treatment. At the same time, strong smoking cessation support (such as counseling, and medication) and effective anxiety management (such as cognitive‐behavioral therapy, and stress relief) should be provided. These strategies aim to identify risks early, break the vicious cycle, and potentially reduce the risk of psoriasis.

The gender stratification analysis in this study found that women may be more sensitive to the impact of anxiety and its duration, especially in the case of combined alcohol consumption. The positive correlation between anxiety days and psoriasis risk is more significant, while no similar association was observed in men. This gender difference may be related to differences in neuroendocrine sensitivity and the immunomodulatory mechanisms of sex hormones. The HPA axis of women is more responsive to psychological stress (such as anxiety) (Duncan et al. 2023; Wright et al. 2023). Alcohol intake can further disrupt the balance of HPA axis function and activate skin mast cells to release pro‐inflammatory mediators (such as IL‐6, TNF‐α, and IL‐17), amplifying neurogenic inflammatory responses and exacerbating the pathological process of psoriasis (Choi et al. 2024; Verplaetse et al. 2024). In addition, sex hormones play a key role in immune regulation (such as T cell function and cytokine secretion) and psoriasis (Cassalia et al. 2025; Marek‐Jozefowicz et al. 2022). Estrogen exerts anti‐inflammatory effects through the estrogen receptor (ER) signaling pathway, including inhibiting Th17 cell differentiation, reducing the expression of pro‐inflammatory cytokines, and enhancing regulatory T cell function (Alanazi et al. 2024; Xie et al. 2025). Low estrogen levels (such as during menstruation and after menopause) weaken this protective barrier, making women more susceptible to anxiety‐induced inflammation (Yoh et al. 2023). Meanwhile, androgens may have a certain anti‐inflammatory effect by inhibiting the expression of pro‐inflammatory cytokines (Ainslie et al. 2024), which may partially explain the weak association in males. However, a decrease in androgen levels or metabolic abnormalities may weaken its protective effect. Future research can combine cues containing detailed hormone data to further validate the specific mechanisms of hormone action in gender differences.

Certain limitations persist in this project. First, we fail to infer a causal relationship between the status and duration of anxiety and the risk of developing psoriasis in this cross‐sectional study. Second, anxiety assessment relies on self‐reported anxiety days in the past 30 days. Although this indicator has population screening validity as a core item of CDC health monitoring (HRQoL), there is still a risk of recall bias. We suggest that future research combine standardized scales (such as GAD‐7) to improve measurement accuracy. Thirdly, NHANES lacks severity grading data for psoriasis, which may affect the interpretation of the results. Finally, confounding factors such as drug treatment were not included, or there may be residual confounding. More research is required in the future to confirm the combined effects of anxiety, smoking, and hypertension on the risk of psoriasis, as well as their potential mechanisms.

## 5 Conclusion

The results indicated that anxiety level and the number of days are positively linked with the risk of psoriasis. Individuals with a combination of anxiety, smoking, and hypertension have a considerably elevated risk of psoriasis. Women, especially those who drink alcohol, are more sensitive to anxiety status and days, with a more significant association between increased anxiety days and increased risk of psoriasis. Since psoriasis cannot be cured, to prevent the disease, people should not only maintain a healthy lifestyle, such as balanced nutrition, regular exercise, reduced smoking, reduced alcohol consumption, and relaxation, but also regularly check blood pressure and lipid levels to prevent abnormalities and maintain cardiovascular health. Women should pay special attention to regulating anxiety while drinking alcohol and regularly monitor blood pressure and cardiovascular health. Furthermore, longitudinal studies should be conducted in the future to confirm the causal association of anxiety and days of anxiety with the psoriasis risk.

## Author Contributions

**Jie Bai**: conceptualization, investigation, funding acquisition, writing – original draft, methodology, writing – review and editing, formal analysis, project administration, data curation. **Yan Ma**: validation, visualization, software, supervision, resources.

## Conflicts of Interest

The authors declare no conflicts of interest.

## Ethics Statement

The authors have nothing to report.

## Peer Review

The peer review history for this article is available at https://publons.com/publon/10.1002/brb3.70817

## Data Availability

Data sharing is not applicable to this article, as no datasets were generated or analyzed during the current study.
